# Experimental and simulation analysis of biogas production from beverage wastewater sludge for electricity generation

**DOI:** 10.1038/s41598-022-12811-3

**Published:** 2022-06-01

**Authors:** Anteneh Admasu, Wondwossen Bogale, Yedilfana Setarge Mekonnen

**Affiliations:** 1 Center for Environmental Science, College of Natural and Computational Sciences, Addis Ababa University, P.O. Box 1176, Addis Ababa, Ethiopia; 2 School of Mechancial and Industrial Engineering, Addis Ababa Institute of Technology, Addis Ababa University, P. O. Box 1176, Addis Ababa, Ethiopia

**Keywords:** Environmental chemistry, Biotechnology, Environmental sciences, Chemistry, Engineering, Materials science, Nanoscience and technology

## Abstract

This study assessed the biogas and methane production potential of wastewater sludge generated from the beverage industry. The optimization of the biogas production potential of a single fed-batch anaerobic digester was operated at different temperatures (25, 35, and 45 ℃), pH (5.5, 6.5, 7.5, 8.5, and 9.5), and organic feeding ratio (1:3, 1:4, 1:5, and 1:6) with a hydraulic retention time of 30 days. The methane and biogas productivity of beverage wastewater sludge in terms of volatile solid (VS) and volume was determined. The maximum production of biogas (15.4 m^3^/g VS, 9.3 m^3^) and methane content (6.3 m^3^/g VS, 3.8 m^3^) were obtained in terms of VS and volume at 8.5, 35 ℃, 1:3 of optimal pH, temperature, and organic loading ratio, respectively. Furthermore, the maximum methane content (7.4 m^3^/g VS, 4.4 m^3^) and biogas production potential (17.9 m^3^/g VS, 10.8 m^3^) were achieved per day at room temperature. The total biogas and methane at 35 ℃ (30 days) are 44.3 and 10.8 m^3^/g VS, respectively, while at 25 ℃ (48 days) increased to 67.3 and 16.1 m^3^/g VS, respectively. Furthermore, the electricity-generating potential of biogas produced at room temperature (22.1 kWh at 24 days) and optimum temperature (18.9 kWh) at 40 days was estimated. The model simulated optimal HRT (25 days) in terms of biogas and methane production at optimum temperature was in good agreement with the experimental results. Thus, we can conclude that the beverage industrial wastewater sludge has a huge potential for biogas production and electrification.

## Introduction

Nowadays, various wastes are sustainably recycled into useful products, for example, energy-efficient brick^1^, packaging^2^, agricultural use^3^, and make different bioenergy systems^4,5^ such as bioethanol^5,6^, biodiesel^7,8^, biogas^9^, and briquette production^10^. To enable the sustainable development of energy supply and mitigate greenhouse gas emissions, biogas production through anaerobic digestion from various feedstocks such as crops, residues, and wastes (industrial, agricultural, and municipal wastes) plays a key role^11^. Biogas production from industrial sludges has several advantages. In addition to sustainable biogas energy production, it has also the advantage of treating organic wastes. Moreover, the development of upgraded biogas techniques will further boost the utilization of biogas for versatile applications including in the cooking and transportation sector^12^. Anaerobic digestion is a sequence of the biological process by which microorganisms convert organic matters into biogas in the absence of oxygen. Biogas is composed of approximately 60 percent methane (CH_4_), 40 percent carbon dioxide (CO_2_), and trace amounts of other gases, for example, water vapor (H_2_O) and hydrogen sulfide (H_2_S). Thus, anaerobic digestion can play a significant role in addressing all of the aforementioned concerns plaguing underdeveloped and developing nations (i.e., energy and waste management) while simultaneously increasing agricultural productivity.

In the previous studies by Ngoc and Schnitzler (2009)^13^ and Goňo et al. (2013)^14^ reported that biogas produced from the fermentation can be combusted to generate combined heat and power (CHP) and lighting during the production processes. Biogas systems with good quality biogas can be used as a source of electricity, which is highly beneficial for environmental protection and development. The effluents from the food and beverage industry are contaminated with toxic metals, which can affect adversely human health as either acute or chronic diseases^15,16^. The millions of gallons of wastewater that pass-through treatment plants each day contain hundreds of tons of biosolids. According to USEPA (1979) report, biosolids generate biogas through anaerobic digestion, which can be produced 55 to 70 percent methane and 25 to 30 percent of carbon dioxide^17^. Nevertheless, biogas production from biomass waste and its utilization for energy applications are still challenging due to the complex physical and chemical properties of organic waste, which affect the metabolic pathways and methane content. Consequently, attention has focused on opportunities for further improvement in biogas yield and quality^18^. Therefore, wastewater sludge is the primary area of research concern in the scientific community, especially in the food and beverage industry. According to Sreekrishnan et al. (2004) report feedstock sometimes requires pretreatment to increase the methane yield in the anaerobic digestion process^16^. Pretreatment breaks down the complex organic structure into simpler molecules that are then more susceptible to microbial degradation. Moreover, the methane yield and content in the biogas can be enhanced by the utilization of chemicals (for example, CaO_2_) during the pretreatment process, it enables further breakdown and degradation of sludge material^19,20^.

The primary factor in determining the methane generation potential of wastewater is the amount of degradable organic material in the wastewater. Properly managed sludge generated from the beverage wastewater treatment plant could potentially yield substantial energy in the form of biogas, potentially turning the wastewater treatment plant into a net energy maker rather than a consumer. In addition to maximizing energy production, the anaerobic digestor enabled the minimization of the total wastewater treatment plant costs. The biogas energy produced from various sustainable feedstocks can be used as a fossil fuel alternative to produce electricity and vehicle fuel. It has the advantage of mitigating greenhouse gas (GHG) emissions from Wastewater treatment plant processes^21^. Labutong et al. (2012)^22^ and Thyo and Wenzel (2007)^23^ suggested an on-the-spot utilization of produced biogas for CHP use without upgrading it to biomethane. Nevertheless, the CHP unit causes direct GHG emissions to the environment and triggers pertinent impacts in the categories of global warming, smog formation, acidification, and eutrophication. The emission rates can be affected by engine type, for example, gas engines with catalytic converters prove the lowest emission rates. Whereas, oil ignition in the pilot injection engines rises the quantities of pollutants in the deplete gas^24^. In general, electricity generation from biogas has lower environmental impacts compared to electricity produced from fossil fuel-based energy systems^24–27^. In this study, combined experimental and computational approaches are employed to estimate biogas production and electricity generation potential from beverage wastewater sludge.

## Materials and methods

### Chemicals

Sulfuric acid (H_2_SO_4_), Manganese sulfate (MnSO_4)_, alkaline azide, starch indicator, 0.02 N Na_2_S_2_O_3_, COD reagent, deionized water, NaOH, benzoic acid(C_6_H_5_COOH), Methyl orange, Sodium carbonate (Na_2_CO_3_), Distil water, KHP (Potassium hydrogen phthalate, HOOCC_6_COOK), Potassium dichromate (K_2_Cr_2_O_3_), Ferrous indicator, mercury Sulfate, FAS (Ferrous ammonium sulfate, Fe(NH_4_)_2_.H_2_O, COD acid, Ammonium solution, or sodium hydroxide, and activated carbon silica gel. The laboratory equipment was used for the study Adiabatic or bob calorimeter, digital weight balance, stirrer, thermometer, electrode, oxygen bomb or vessel equipment, oxygen hose, resister, capsule, cotton thread, or fuse wire, bucket, funnel, volumetric flask, EA1112 flash CHNS/O-analyzer and BOD incubator.

### Characterization of substrate

#### Proximate analysis of substrate

Proximate analysis is the determination of the total solid content, volatile solids, moisture content, fixed carbon, sulfur, and ash content. The dry solid may be defined as the mass of material remaining after heating the substrate to 105 ℃ for 1 h expressed as a percentage of the mass of the starting wet material. According to Murphy et al. 2015^28^ the volatile solid content may be defined as the mass of solid lost during ignition at 950 ℃ for 7 min in a covered crucible expressed as a percentage of dry solid. BOD was determined using the standard HACH method. COD was determined by using AL 450 AQUALYTIC photometer with SN 11/4005 made in Germany’s standard measurement method. The determination of energy content of the wastewater sludge was determined using a bomb calorimeter. Phosphate could be determined by using APHA 4500-P-C molybdate acid colorimetric method.

#### Ultimate analysis

The ultimate analysis assesses the portion of carbon, hydrogen, sulfur, and nitrogen in a dry solid sample of the substrate. Therefore, for this study, the ultimate analysis was carried out in the conditions of a gas flow rate of 120 ml/min, a reference flow rate of 100 ml/min, an oxygen flow rate of 250 mL/min, furnace temperature of 900 °C, and oven temperature of 75 °C. The six-calibration point for every component and sample was run in induplicate.

To our knowledge, no study has been reported yet for the production of biogas from soft drink beverage industrial waste sludge. In line with this, our preliminary survey in a single fed-batch anaerobic digester with a 1:1 ratio of water and wastewater sludge with a total volume of 20 L water jars has shown a higher yield of biogas composition (61.11% CH_4_). With this motivation, this study was focused on the characterization of Physico-chemical analysis of wastewater sludge, optimizing different variables (temperature, pH, organic loading ratio, and hydraulic retention time), and optimizing different variables with software simulation for biogas production.

#### Estimated potential for methane electricity generation

Producing biogas through anaerobic digestion is important for maximizing energy production and lowering overall treatment costs in WWTPs. The use of biogas for power and fuel as opposed to natural gas has numerous environmental benefits, including a lower carbon footprint. Similarly, biogas should be used for on-site CHP rather than upgrading to biomethane to maximize GHG mitigation. In general, biogas-generated electricity has a lower environmental impact than fossil-fuel-generated electricity^21–23,25^. The study was asses to estimate the amount of biogas and electricity generated from beverage wastewater sludge. The simplest way to generate electricity from biogas is with an internal combustion engine, and the amount of electricity produced from biogas can be calculated using the equation below^24–27,29^.

$${E}_{elec}={Q}_{biogas}*{F}_{CH4}*{CP}_{CH4}*{\eta }_{elec}$$

where: ${E}_{elect}$ is the electrical energy produced per tonne of organic residues (tres), in KW/tres, ${Q}_{biogas}$ is the amount of biogas obtained from the organic residues of a biodigester, in m^3^, ${F}_{{CH}_{4}}$ is the methane contained in the biogas, in percentage, ${CP}_{{CH}_{4}}$ is the specific heat of methane (KWh/m^3^), ${\eta }_{elect}$ is the electrical efficiency in percentage.

The anaerobic digestion process as well as the precise chemical composition of the organic waste, which varies depending on the waste collection point, affects ${Q}_{biogas}$ and ${F}_{{CH}_{4}}$. The precise ratio of CH_4_ to CO_2_ in biogas is determined by the type and concentration of organic input, which serves as the feedstock for the microorganisms at work during the anaerobic and fermentation processes. Anaerobic digestion is a well-established waste and wastewater treatment technology^27^.

#### Waste calorific value estimation

The high heating value (gross calorific or gross energy value) is defined as the amount of heat released by a given quantity after it has been combusted and the products have returned to a temperature of 25 °C. A low heating value (LHV, net calorific value) is defined as the amount of heat released by combusting a specified amount when the final temperature of the combustion products is greater than the boiling point of water (100 °C). The LHV assumes the latent heat of vaporization of water in the fuel and does not recover the reaction products. The high heating value accounts for the latent heat of water vaporization in the combustion products. In general, the two terms of calorific values, High Heating Value and Low Heating Value, were used to describe the heat contents. The high heat value and low heat value of waste are calculated using the Dulong Equation, which is shown below. To determine a fuel's LHV from its HHV or vice versa, the moles of water produced when a mole of fuel is burned must be determined^29^.

$$HHV\left(\frac{kJ}{kg}\right)=33801\left(C\right)+144158 \left\{\left(H\right)-0.125\left(O\right)\right\} +9413(S)$$

$$HHV=LHV+n\Delta Hv ({H}_{2}O,25^\circ{\rm C} )$$

where carbon, hydrogen, oxygen, and sulfur are the C, H, O, and S content (dry basis).

The heat of vaporization of water at 25 ℃ is;

$$\Delta HV\left({H}_{2}O,25^\circ{\rm C} \right)=\frac{44.013kJ}{mol}or 2.445\frac{\mathrm{MJ}}{\mathrm{kg}}.$$

### Experimental setup of optimization

**Figure 1:**
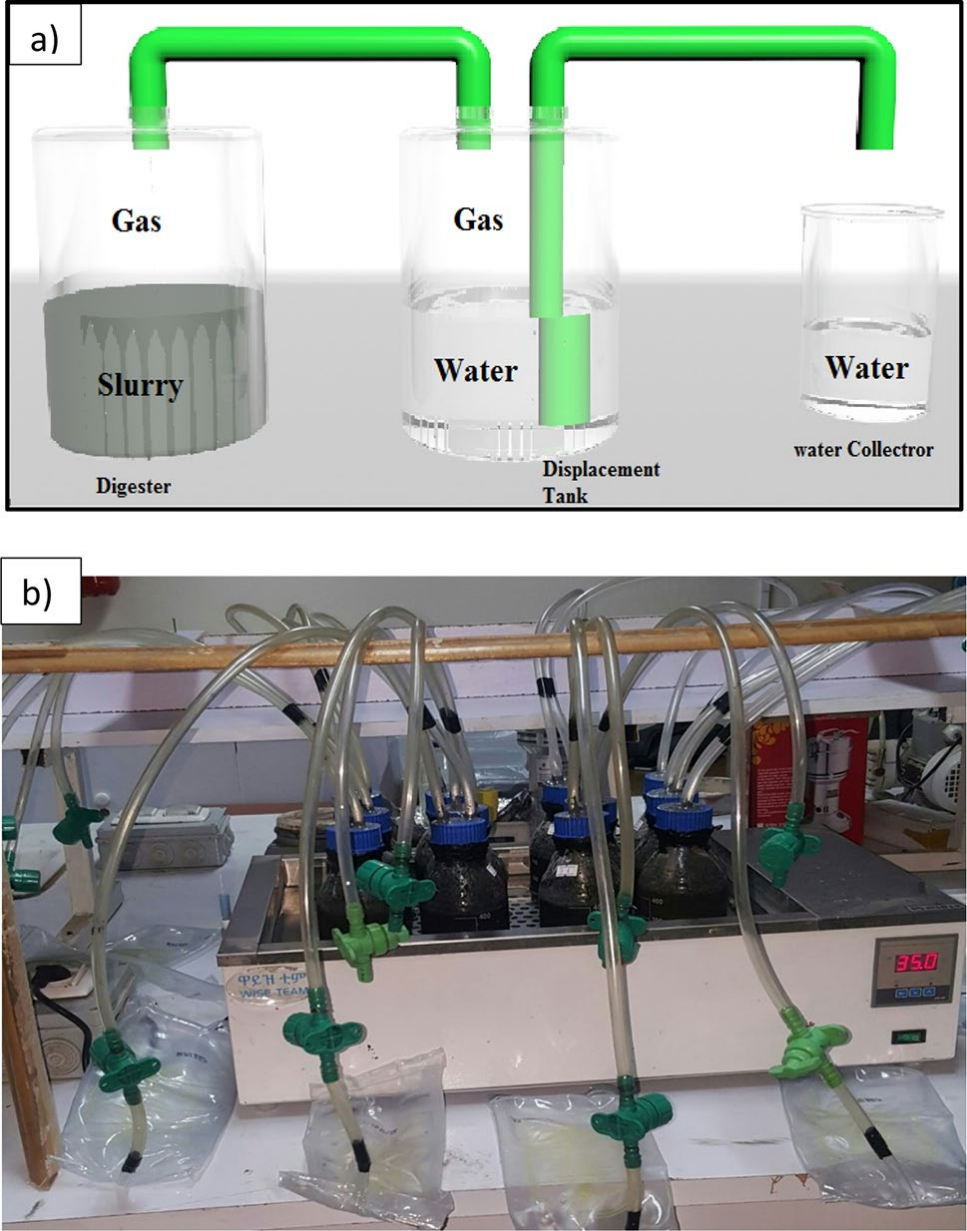
HYPERLINK "sps:id::fig1||locator::gr1||MediaObject::0" (**a**) Schematic diagram of the experimental setup for small-scale anaerobic digestion. (**b**) Experimental setup for biogas production on a laboratory scale.

According to the method described by Wong et al. (2011)^30^ anaerobic batch digestion pH, organic loading rate, temperature, and hydraulic retention time optimization tests were carried out in triplicate and incubated in a water bath at 35 ℃. Experiments were carried out for 30 days to describe the beginning of biogas production that is necessary to determine the optimum HRT of biogas potential. The batch digester is sealed from the inside to prevent biogas leakage and a fully inserted water bath to maintain temperature^13^. The contents were stirred by using handshaking. For this study, biogas production was measured monthly by using an air tie syringe and biogas composition was collated by using gas analyzer equipment. According to Sreekrishnan et al.^16^ 1% NaOH and H_2_SO_4_ were used to adjust the pH of the substrate. The optimization of biogas production is carried out by collecting wastewater sludge and different laboratory equipment. The following laboratory equipment was used: water bath, reactor bottles, gas regulator valve, plastic hose, air tie gas syringe, gas analyzer, and gas collector bags. The experimental setup of biogas production optimization in small-scale anaerobic digestion and laboratory-scale are shown in Fig. 1a,b, respectively.

### Kinetic model of anaerobic digestion

In this study, the kinetic model of an aerobic digestion was used to estimate the quantity of methane from the produced biogas. In 1936, Buswell and Hatfield developed the stoichiometric formula that enables the prediction of methane content from the produced biogas in 1936^31,32^. Later in 1952, Boyle modified the chemical reaction of Buswell and Mueller to enable the nitrogen and sulfur are included to obtain the fraction of NH_3_ and H_2_S in the produced biogas^32–35^. MATLAB software was used for simulation analysis of biogas production from wastewater sludge. Industrial wastes are very complex mixtures and different approaches are used to describe their composition. The composition of the elements is the most useful basic method to describe the non-aqueous components of the wastewater sludge. The goal of this model is to provide a balance between simplicity and effective biogas production prediction. The purpose is not considered to create a model that considers all factors and predicts biogas output to a very high level of precision. Therefore, this simple model study is used in order to estimate the theoretical biogas potential. To apply this model to a specific feedstock, we need to know the chemical components of the feedstock. The model was considering the assumption of the input material consists of only carbon, hydrogen, oxygen, nitrogen, and sulfur elements. The relative ratios of these elements can be taken from the ultimate analysis of the waste, constant temperature, constant digester volume, perfect mixing, ideal bacterial condition, and products of the reaction including only CH_4_, CO_2_, NH_3,_ and H_2_S. No accumulation of ash and the reaction goes to completion^34^.

## Results and discussion

### Characteristics of substrate

The main part of biogas is carbon, oxygen, hydrogen, and sulfur. The quantity and quality of product derived from any waste to the energy conversion process. The ultimate analysis of the substrate was used to determine the stoichiometric equation based on the elemental composition of waste material and to calculate the theoretical methane composition by taking into account C, H, O, and N^36^. From this study, the dry wastewater sludge contained about 45.190% mass of carbon and about 42.992% mass oxygen on a dry weight basis of the wastewater sludge. The ultimate characteristics of wastewater sludge are shown below in Table 1.

**Table 1:** The ultimate analysis result of beverage wastewater sludge.

Types of the element	The ultimate value of the element (%)
C	45.19
N	3.579
H	7.299
S	0.94
O	42.992

According to the proximate result, the dried waste sludge showed a moisture content of about 6.26%. The remaining fraction of mass contents is the total solid content present in wastewater sludge. This solid mass largely contains volatile solids and a little fraction is as mineral contents (ash). In any energy conversion process, only a part of volatile solids mass undergoes its conversion. The proximate properties of the wastewater sludge are summarized in Table 2.

**Table 2:** Proximate analysis of the beverage wastewater treatment sludge.

Sample	Moisture (%)	Volatile solid (%)	Ash (%)	Fixed carbon (%)	Sulfur (%)
Sludge	6.3	60.1	27.0	6.7	0.9

According to Fytili and Zabaniotou (2008)^37^ and Sitorus et al. (2013)^38^ reported heating values for several types of sewage sludge in the range of 11 to 25.5 MJ/Kg (2627 to 6000 cal/g). Moreover, Oladejo et al. (2019)^39^ reports the volatile organic contents of dried sewage sludge ranges from 21–48%, as a consequence, the energy content varies between 2600 and 5200 cal/g. In this study, the calorific value of the sludge from the beverage wastewater treatment plant was about 5042.2 cal/g, which is in good agreement with the higher heating values of the above literature. According to previous related works done in the ultimate analysis, the amount of carbon content (W%) is directly proportional to the calorific value^40–42^. This means that if the sludge contains a lot of carbon, it also contains many calories. Our substrate has a high carbon content, according to the ultimate analysis (45.19%). This is because sugar is one of the most important basic materials in the soft drink beverage industry. As a result, the high calorific content of our substrate is most likely due to the raw materials used in the soft drink beverage industry. In addition, the phosphate, TS, COD, and BOD volume are 4.02 mg/l, 27.4%, 2200 mg/l, and 30 mg/l, respectively. The phosphorous concentration was determined based on the external calibration curve with a good coloration factor (R^2^ = 0.999).

### Biogas production from wastewater sludge at laboratory scale

**Figure 2:**
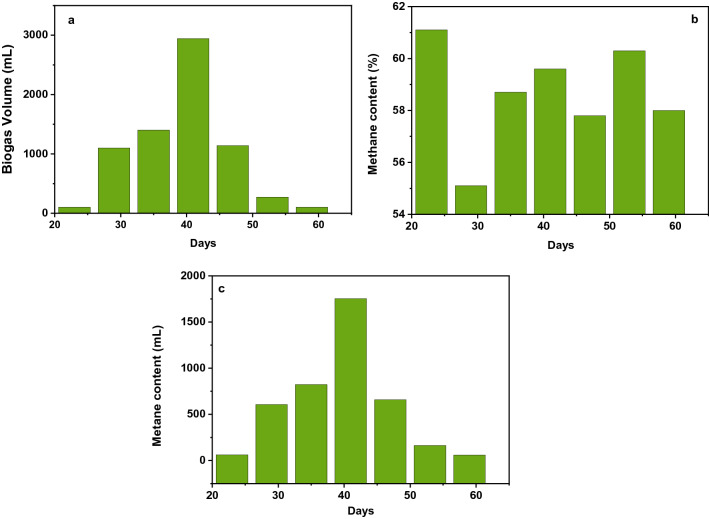
(**a**) Biogas production (mL) as a function of days, (**b**) methane content (%) as a function of days, (**c**) methane content (mL) as a function of days on a laboratory scale for preliminary study.

In this research, a single fed-batch anaerobic digester with a total volume of 20 L water jars was used for the production of biogas. The feedstock contains 50% of wastewater substrate and 50% of water. The total weight of feedstock loading was 20 kg and mixed manually during the feeding. It was operating at environmental conditions without any parameter control. The gas collector bag was provided for a collection of biogases. The production of biogas was determined by using the water displacement method periodically and analyzing biogas composition by using a gas analyzer. From this primary assay experiment study, biogas production was started after a 23 hydraulic retention time. Figure 2 shows the result of the biogas production volume and methane content of this study.

### Optimization of biogas production variables

#### pH

**Figure 3:**
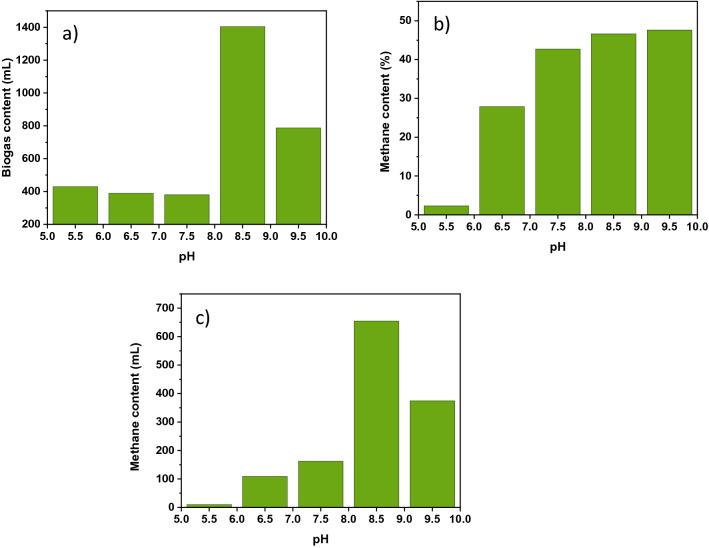
(**a**) Biogas production (mL) as a function of pH, (**b**) methane content (%) as a function of pH, (**c**) methane content (mL) as a function of pH.

pH optimization was conducted at constant substrate ratios and temperature were kept at 1:4 and 35 ℃, respectively for all the experimental setups. The optimization was performed in triplicate analysis. Each reactor had a 500 mL capacity and contained 400 mL of total liquid, including wastewater sludge substrate. The pH optimization was done in different batch reactor setups as follows. In setup A: reactors 1, 2, and 3 were done at pH 5.5. In setup B: reactors 4, 5, and 6 were done at pH 6.5. In setup C: reactors 7, 8, and 9 were done at pH 7.5, in setup D: reactors 10, 11, and 12 were adjusted at pH 8.5, and the last setup E: reactors 13, 14, and 15 were regulated at pH 9.5. Various researchers reported the range of pH for suitable anaerobic wastewater sludge digestion. The optimal pH for industrial organic waste was obtained between 6.5 and 7.5^43^. According to the previous reports by Rosenberg and Kornelius (2017)^44^, the optimal pH value for biogas production was found to be between 6.7 and 7.5. The study reported by Ngoc and Schnitzer (2009)^13^ also identified that the optimal pH value of anaerobic digestion for biogas production is between 6.0 and 8.0. In this study, the maximum biogas and methane yield was attained at initial pH of 8.5 and the gas production ended at pH 7.3 with a reactor temperature of 33 ℃. Moreover, the maximum biogas yield (1404.3 mL) and methane content (654.4 mL) are presented in Fig. 3, which shows the sharp decline of methane content after pH 8.5.

#### Organic loading ratio

**Figure 4:**
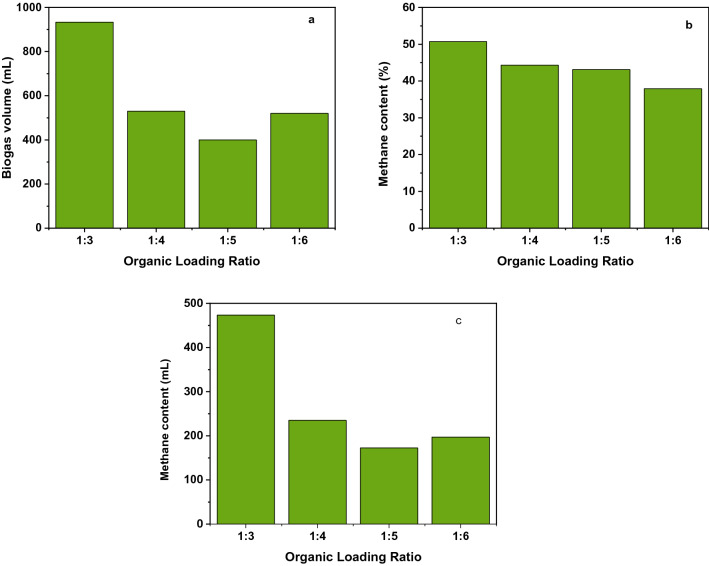
(**a**) Biogas production (mL) as a function of organic loading ratio, (**b**) methane content (%) as a function of organic loading ratio, and (**c**) methane content (mL) as a function of organic loading ratio.

The organic loading ratio optimization was conducted at a constant pH (8.5) and temperature (35 ℃) of the substrate at all experimental setups. Each reactor had a 500 mL capacity and contained 400 mL of total liquid including wastewater sludge substrate. The substrate ratios to water in different batch reactor setups were done as follows. In setup A: reactors 1, 2, and 3 were done in the ratio of the substrate at 1:3. In setup B: reactors 4, 5, and 6 were done with a ratio of the substrate at 1:5. In setup C: reactors 7, 8, and 9 were done with the ratio of substrates at 1:6. In the optimization of organic loading, measurements were carried out in triplicates using three reactors for each organic loading considered in this study. Figure 4 shows the optimum biogas production and methane yield result was measured at a ratio of 1:3. This result is in agreement with the work of Syaichurrozi and Sumardiono (2013)^45^.

#### Temperature

**Figure 5:**
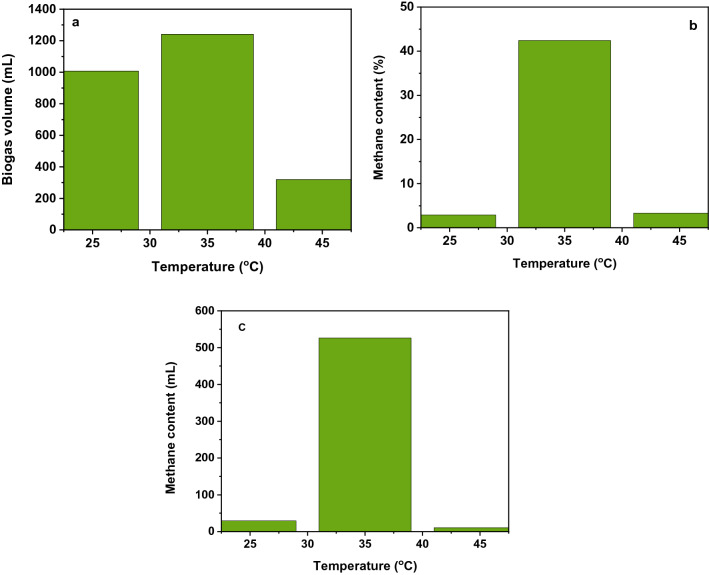
(**a**) Biogas production (mL) as a function of temperature, (**b**) methane content (%) as a function of temperature, and (**c**) methane content (mL) as a function of temperature.

Temperature optimization was conducted at a constant loading ratio, pH of feedstock was kept at 1:3, and 8.5 ratios, respectively at all experiment setups. The reactors were protected in the water baths at different temperatures. The experimental setup at different temperatures has been used: water bath A: 25 ℃, water bath B: 35 ℃, and water bath C: 45 ℃. For each temperature, the experiments were done in triplicates. Furthermore, the maximum yield of methane gas content and biogas production volume at a water bath temperature (35 ℃) and reactor temperature (32 ℃) results are shown in Fig. 5. This result is in good agreement with the literature value reported in the following works^46–49^.

#### Hydraulic retention time

**Figure 6:**
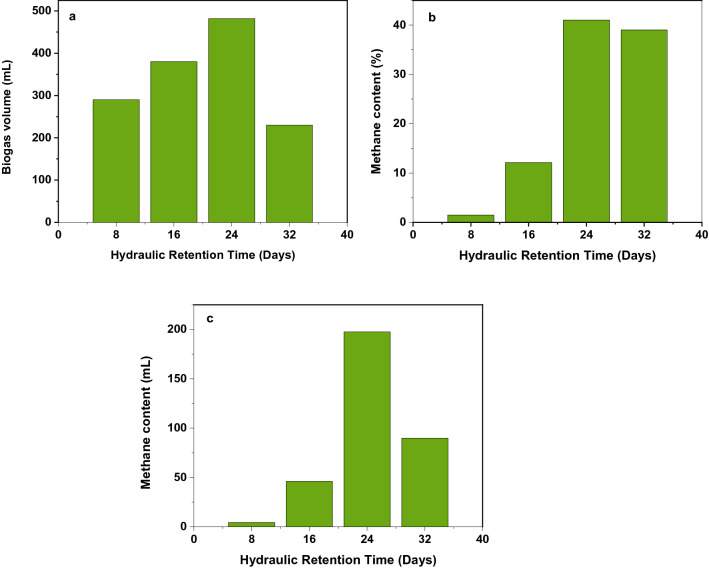
(**a**) Optimal biogas production (mL) as a function of hydraulic retention time, (**b**) optimal methane content (%) as a function of hydraulic retention time, (**c**) methane content (mL) as a function of hydraulic retention time at 35 °C.

The optimal hydraulic retention time was optimized at the optimal temperature, pH, and loading ratio of feedstock. The optimal temperature, pH, and loading ratio of the feedstock were 35 ℃, 8.5, and 1:3, respectively for all experimental setups. Similar to previous reports, the HRT values were measured periodically for 30 days within seven days intervals. According to Rosenberg and Kornelius^44^, Bouallagui et al.^50^, and Ngoc and Schnitzer^13^, the effective AD of organic matter under mesophilic conditions was obtained at 20, 25, and 28–35 days. Moreover, Atelge et al.^51^, reported the optimal HRT range of 20–30 days, respectively. Similarly, in our study, the maximum biogas and methane content obtained at 24 days (Fig. 6) are in good agreement with the above literature values.

**Figure 7:**
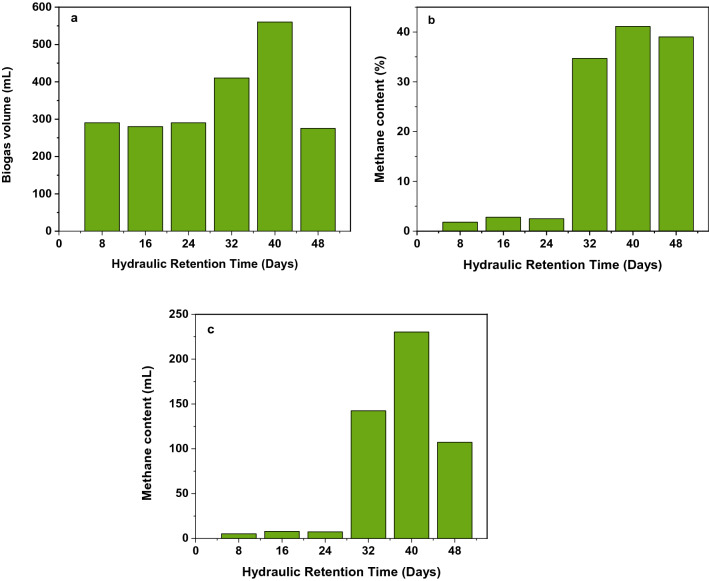
(**a**) Optimal biogas production (mL) as a function of hydraulic retention time, (**b**) optimal methane content (%) as a function of hydraulic retention time, (**c**) methane content (mL) as a function of hydraulic retention time at 25 °C.

The optimal hydraulic retention time was optimized in the psychrophilic bacterial temperature zone (25 ℃) and at optimal pH and organic loading a ratio of 8.5 and 1: 3, respectively. For biogas production, in the comparison of HRT between mesophilic and psychrophilic bacterial temperature zone, the optimal HRT of psychrophilic temperature was longer than mesophilic temperature zone. All experiments were performed in triplicates. The maximum biogas volume and methane content are measured at an optimal HRT of 45 days at a temperature of 25 ℃. The results of maximum biogas volume and methane content at optimal HRT are shown in Fig. 7.

#### Electrical energy potential estimation

According to Davis et al. (2016)^29^ the relation to the specific heat of methane (also known as net heating value or lower heating value), puts Cp(CH_4_) at 10 kWh/m^3^, whilst the Swedish Gas Centre^52^ put Cp(CH_4_) at 9.97 kWh/m^3^. In this study, Cp (CH_4_) = 10 kWh/m^3^ was used. In addition, the electrical efficiency (η_elec_) value depends on the technology used. The efficiency varies between 25 and 31 percent, but where certain technologies are capable of up to 43%. According to the above mention article, the values range from 25 to 40%, but where the majority of technologies presented have minimum efficiencies of 30%. As such, for this study, a value of η_elec_ = 30% is deemed reasonable. For optimizing biogas production, the maximum estimated electricity energy was 18.9 kWh at 24 days, whereas at room temperature of biogas production the maximum estimated electricity energy was 22.1 kWh at 40 days. Likewise, for the water displacement method, the methane content is upgrading up to 61.6%. Based on this result, the electricity production potential from biogas production was shown in incensement and the estimation value was 33.1 kWh at 48 days. However, the total electricity energy potential estimation from biogas production at optimization and room temperature was 54.5 kWh/month and 83 kWh/48 days, respectively.

#### Kinetic model simulation

**Figure 8:**
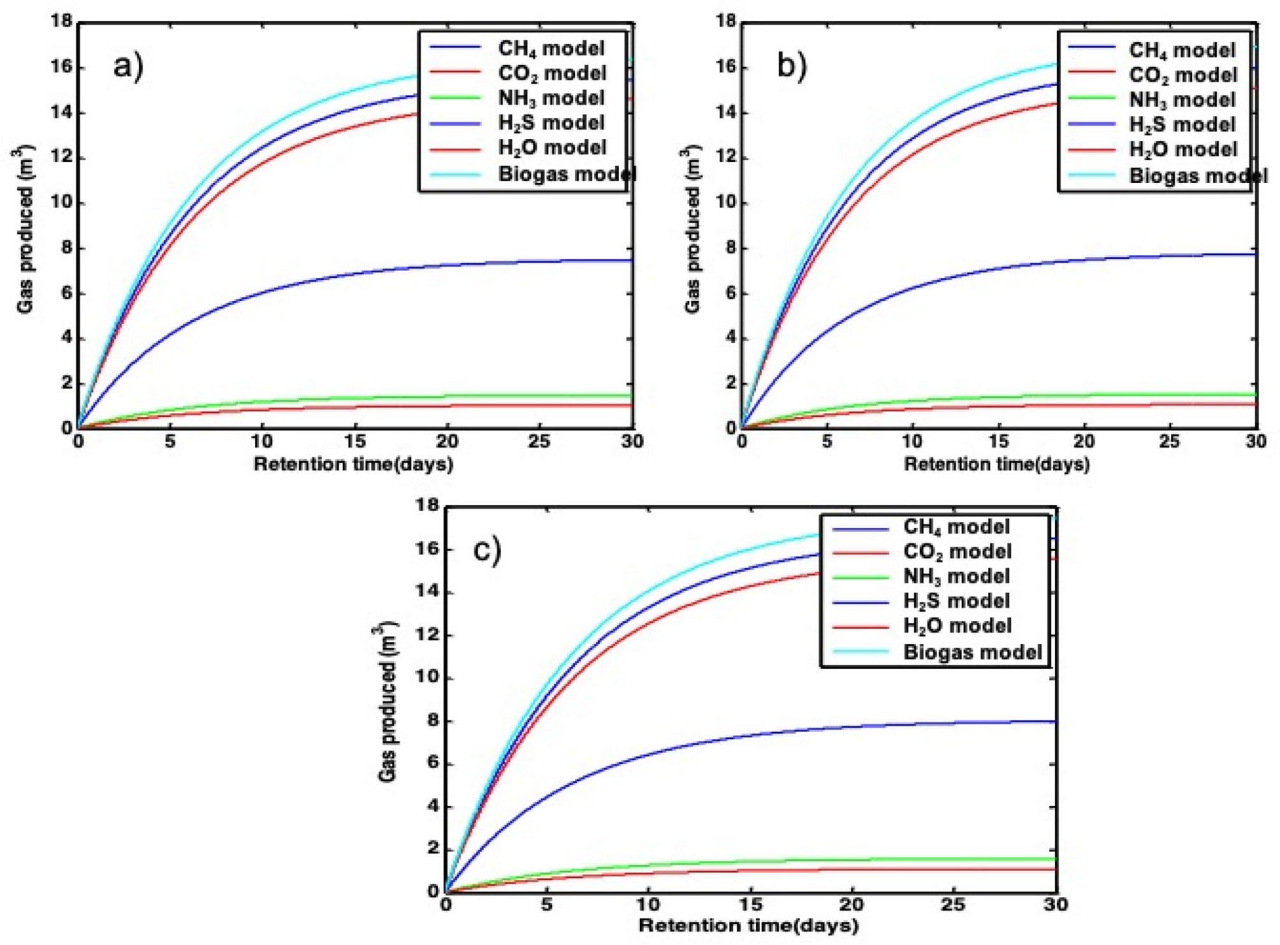
Simulation results of biogas production as a function of retention time (days) at various temperatures of (**a**) 45 ℃, (**b**) 35 ℃, and (**c**) 25 ℃, respectively.

Before optimizing the optimal value of HRT of biogas production, the optimal value of HRT was simulated by using a computer circulation program of an optimization model. The parameters in this model were the same as the experiment parameters mentioned above. It has been observed that the optimal value of HRT for biogas production was reported at different temperature zones, under psychrophilic (25 ℃), mesophilic (35 ℃), and thermophilic (45 ℃), respectively. In the model simulation, an optimal HRT of biogas production was predicted before optimizing the temperature and HRT experimentally. The effects of temperature and HRT for biogas production are predicted in the model simulation of biogas production. In general, regarding temperature and HRT for model simulation biogas production, the temperature of 35 ℃ is the optimal temperature rather than 25 and 45 ℃. Therefore, the optimal temperature of biogas production in model simulation is good agreeing with the experimental result. But, the comparison between model simulation and experimental biogas production at a temperature of 45 was impossible. For this research, biogas cannot produce at a temperature of 45 ℃. Figure 8 shows the kinetic model biogas production results in various temperatures.

#### Comparison between simulation and experimental result

**Figure 9:**
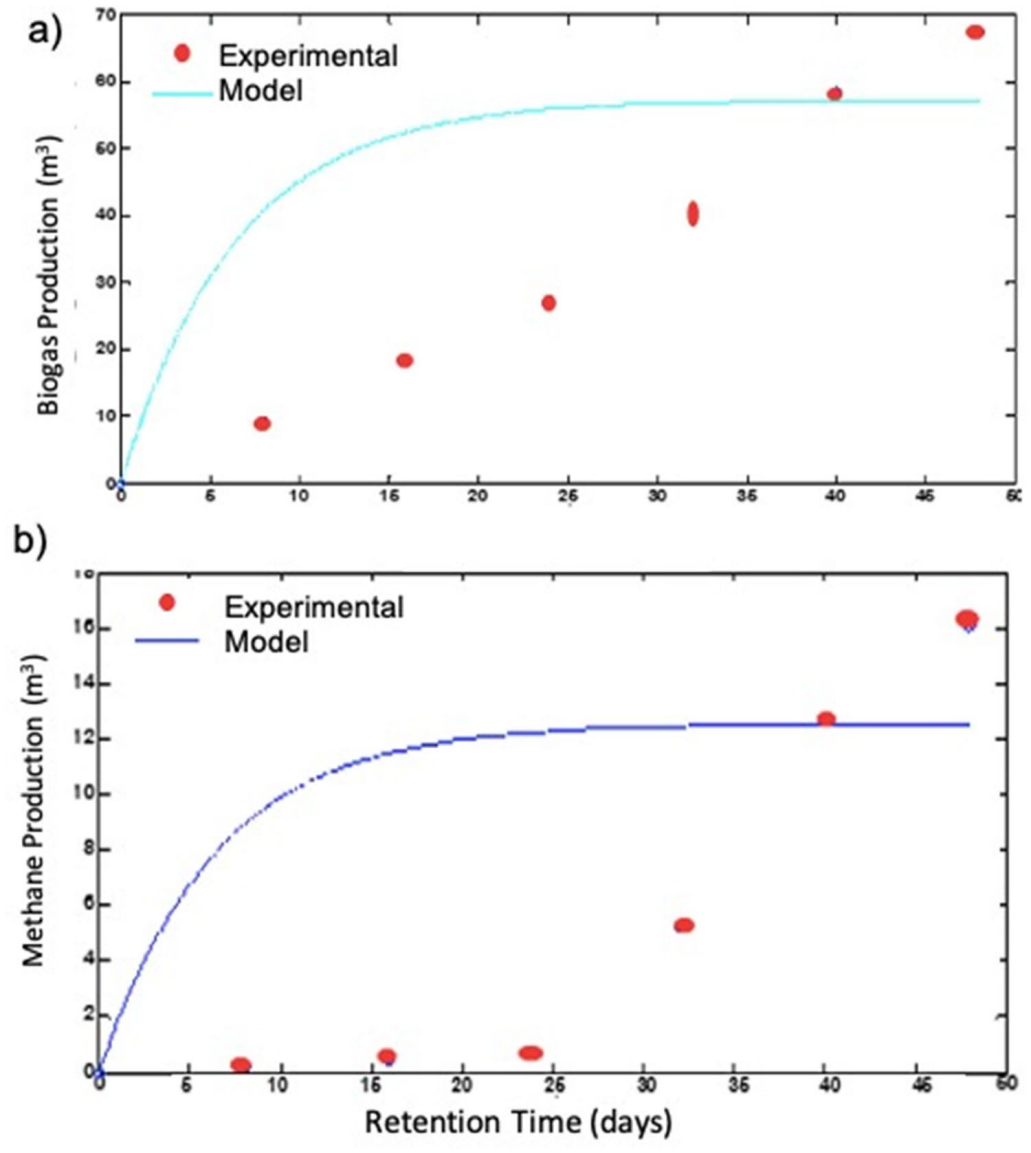
(**a**) Biogas production (m^3^) as a function of time (days), simulation and experimental results, (**b**) methane gas production (m^3^) as a function of time (days), simulation and experimental results at 25 ℃.

The experiments were run under different temperatures, HRT, pH, and organic loading concentrations to determine the optimal parameters of biogas production. The optimal HRT for model simulation biogas production results at a temperature of 25 ℃ are shown for 30 days, but at this temperature, the experimental biogas production result is shown for 40 days. This comparison between model simulation and experimental biogas production results at a temperature of 25 ℃, the model simulation biogas production is less than by 10 days HRT. The optimal HRT between model simulation and experimental methane gas production is 40 days. This result shows similar optimal HRT between simulation and experimental methane gas production, and the comparison between the experiments and model simulation of methane gas production is in good agreement. Moreover, the comparison between experimental and model simulation biogas and methane production result at a temperature of 25 ℃ is shown in Fig. 9.

**Figure 10:**
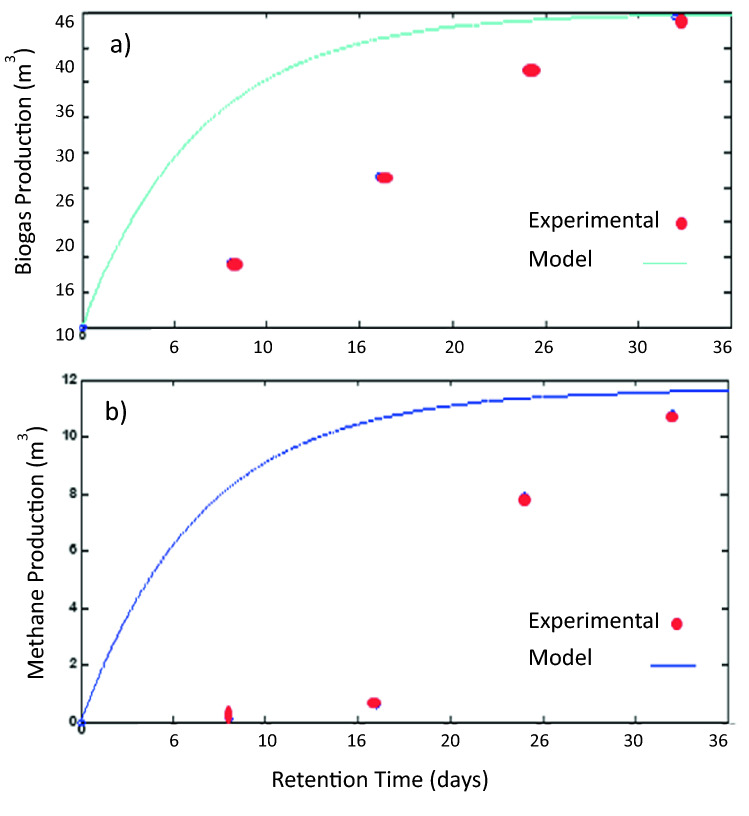
(**a**) Biogas production (m^3^) as a function of time (days), simulation and experimental results, (**b**) methane gas production (m^3^) as a function of time (days), simulation and experimental results at 35 ℃.

The model simulation biogas and methane gas production at a temperature of 35 ℃ the optimal HRT is 25 days. The experimental biogas and methane gas production at a temperature of 35 ℃ the optimal HRT are shown for 24 days. This result is shown the comparison between model simulation biogas and methane gas production is approximately similar. Moreover, the comparison between experimental and model simulation biogas and methane production result at temperatures of 35 ℃ is shown in Fig. 10.

## Conclusion

This paper quantitatively presented the production of electricity from the biogas produced from beverage wastewater sludge at optimal experimental conditions (i.e., temperature, loading ratio, and pH of 35, 1:3, and 8.5, respectively). The experimental results were compared with the model simulation outputs for validation. The maximum methane content of the biogas in terms of VS and volume is 6.3 m^3^/g VS and 3.8 m^3^, respectively at 24 days. The biogas production potential in terms of VS and volume is 15.4 m^3^/g VS and 9.3 m^3^ volume of biogas at 24 days, respectively. Even at room temperature (25 ℃) notable methane content was produced, the maximum methane content of the biogas in terms of VS and volume is 7.4 m^3^/g VS and 4.4 m^3^ CH_4_ at 40 days, respectively. In addition, the biogas production potential at room temperature in terms of VS and volume is 17.9 m^3^/g VS and 10.8 m^3^ volume of biogas at 40 days, respectively. The prediction of optimal temperature and HRT between the model simulation and experimental biogas production is in good agreement. The electricity potential estimation and biogas production at room temperature is 22.1 kWh and 18.9 kWh at 40 and 24 days, respectively. Also, the total electricity generation potential was found to be 83.0 kWh per 48 days and 54.5 kWh per month, respectively. Moreover, employing the water displacement method, enhanced the methane content of the produced biogas to 61.6%, as a consequence, the electricity production potential increased to 33.1 kWh at 48 days. In general, the results of this study revealed that the beverage wastewater sludge could be a very promising feedstock for electricity generation from anaerobic digestion biogas production and methane content upgrading. It plays a vital role in mitigating greenhouse gas emissions and provides cost-efficient and sustainable energy to the industry’s internal consumption and the surrounding community.
